# Chronic alcohol-related myopathy: a closer look at the role of lipids

**DOI:** 10.3389/fphys.2024.1492405

**Published:** 2024-11-18

**Authors:** Muni Swamy Ganjayi, Thomas A. Krauss, Craig R. G. Willis, Cory W. Baumann

**Affiliations:** ^1^ Department of Biomedical Sciences, Heritage College of Osteopathic Medicine, Ohio University, Athens, OH, United States; ^2^ Ohio Musculoskeletal and Neurological Institute, Ohio University, Athens, OH, United States; ^3^ School of Chemistry and Biosciences, Faculty of Life Sciences, University of Bradford, Bradford, United Kingdom

**Keywords:** adipose tissue, atrophy, metabolism, skeletal muscle, weakness

## Abstract

Chronic alcohol-related myopathy (CAM), characterized by muscle atrophy and weakness, arises from prolonged excessive ethanol (EtOH) intake. The precise mechanisms by which EtOH induces skeletal muscle atrophy are not fully understood. This article posits that the pathophysiology of CAM may be significantly influenced by how EtOH modifies lipid profiles and alters lipid composition and content in skeletal muscle. We review existing literature on lipid alterations in CAM-afflicted individuals and analogous animal models, discuss EtOH’s direct and indirect effects on skeletal muscle lipids, and present specific instances where lipids contribute to muscle atrophy. This article advocates for a novel viewpoint, suggesting that lipid dysregulation may be the principal factor in EtOH-induced muscle wasting, offering a different angle to approach CAM research and treatment strategies.

## Introduction

Chronic consumption of alcohol leads to various histological, biochemical, and physiological changes in skeletal muscle (Lang et al., 2005). These changes can result in chronic alcohol-related myopathy (CAM), a disorder marked by muscle wasting and weakness, particularly in fast-twitch muscles (Preedy et al., 2001). The degree of skeletal muscle atrophy correlates with the amount of alcohol consumed over a lifetime, potentially eroding up to 20% of total muscle mass and significantly reducing strength under extreme conditions (Ekbom et al., 1964; Rossouw et al., 1976; Martin et al., 1985; Estruch et al., 1998; Aagaard et al., 2003). Affecting approximately 40%–60% of chronic alcoholics, CAM is more prevalent than alcohol-induced liver cirrhosis yet remains under-researched (Preedy et al., 2003). The intricate pathophysiological mechanisms that contribute to the development and progression of CAM are still to be fully elucidated.

Preclinical studies in rodents have enhanced our understanding of CAM. Rodent studies, utilizing various methods of ethanol (EtOH) administration (such as liquid diet or drinking water), consistently demonstrate significant muscle atrophy and weakness following prolonged EtOH exposure (Lang et al., 1999; Crowell et al., 2019; Moser et al., 2022; Moser et al., 2023; Ganjayi et al., 2023). These effects are observed independently of caloric intake and other influential factors like diet and lifestyle, suggesting an obvious link between EtOH and CAM. Historically, research has concentrated on EtOH’s role in diminishing muscle mass via impeding protein synthesis via mTORC1 signaling (Preedy et al., 1991; Lang et al., 2001; Steiner and Lang, 2015; Simon et al., 2023). However, these studies narrow the focus on this single mechanism, potentially neglecting other contributing or upstream factors. For this article, we suggest that the onset and progression of CAM might also be attributed to EtOH’s direct or indirect effects on skeletal muscle lipids.

### Impact of chronic EtOH consumption on skeletal muscle lipid profiles

Chronic EtOH consumption has a profound impact on lipid composition and metabolism in skeletal muscle, as evidenced by a series of studies spanning several decades. To our knowledge, the earliest known investigation by Sunnasy et al. (1983) revealed that chronic alcoholics (at least 100 g of EtOH daily for 3 years) with myopathy had a 53% increase in total lipid content in the quadriceps muscle, primarily due to triglycerides. This was characterized by elevated levels of palmitate (16:0), oleate (18:1), and arachidate (20:0), and lower levels of myristate (14:0), stearate (18:0), and linoleate (18:3).

Further research in male Wistar rats demonstrated that 6 weeks of EtOH consumption (75 mmol/kg body weight) altered fatty acid composition in soleus and plantaris muscles, with linoleic (18:2) and oleic (18:1) fatty acids increasing and decreasing, respectively (Salem et al., 2006). This was complemented by others (Kulagina et al., 2018) who demonstrated a duration-dependent response in the gastrocnemius muscle of male Wistar rats that consumed a 10% aqueous EtOH solution plus a 30% EtOH solution in agar blocks. After 12 weeks, myristic, vaccinic, dihomo-γ-linolenic, ω-6-decosapentaenoic, palmitic, palmitoleic, oleic, and linoleic fatty acids increased (or tended to increase), whereas at 24 weeks total fatty acid content was lower, with myristic (14:0), oleic (C18:1, ω-9), linoleic (C18:2, ω-6), α and γ linolenic (c18:3, ω-6, ω-3), eicosadienoic (C20:2), and polyunsaturated fatty acids all decreasing.

A more recent study using the gastrocnemius muscle from male C57BL/6 mice consuming EtOH that accounted for 27.5% of total calories for 4 weeks provided additional insights (Zhao et al., 2011). Zhao et al. (2011) found that while total lipids remained unchanged, individual lipids containing 18:3, 18:2, 18:1, and/or 18:0 fatty acids increased 41%–152%, whereas levels of 16:0/20:4 phosphatidylcholines (PC) and 16:0/22:6 PC decreased 29%–35%. Furthermore, Koh et al. (2020) reported that triglycerides accumulated in skeletal muscle after 4 weeks of 5% EtOH intake (specific muscle and sex of the mice were unspecified). These clinical and preclinical findings collectively underscore the significant regulatory effects of EtOH on the skeletal muscle lipid profile, which has implications for understanding the pathophysiology of alcohol-related muscle disorders and the development of therapeutic strategies.

### Mechanisms by which EtOH may be altering skeletal muscle lipids

**FIGURE 1:**
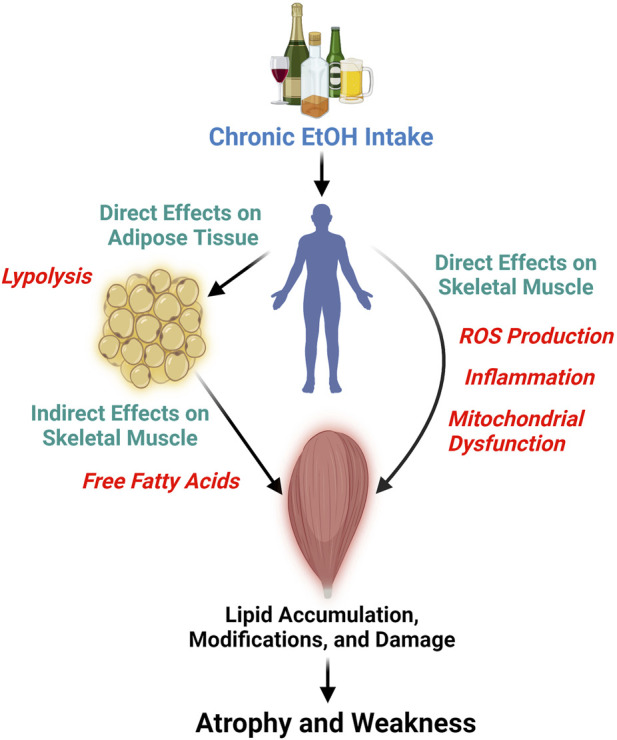
This diagram illustrates the detrimental effects of chronic and persistent alcohol (ethanol, EtOH) consumption on skeletal muscle, leading to atrophy and weakness. It highlights the direct impact of EtOH on skeletal muscle lipids through the generation of reactive oxygen species (ROS), triggering inflammation, and mitochondrial dysfunction, which are likely interconnected processes. Furthermore, the figure depicts the indirect effects of EtOH on skeletal muscle lipids, mediated by its action on other tissues, such as adipose tissue. Here, EtOH induces lipolysis in adipocytes, elevating the levels of free fatty acids that may subsequently infiltrate skeletal muscle. Figure was created using BioRender.

Chronic EtOH consumption appears to affect muscle tissue lipid profiles through direct and indirect pathways (Figure 1). Directly, EtOH can alter lipid profiles via several interrelated mechanisms: generation of reactive oxygen species (ROS), triggering of a proinflammatory response, and disrupting mitochondrial function. For instance, the activity of the glutathione peroxidase system is known to decrease in chronic alcoholics and in rodent models of excessive EtOH consumption (Guerri and Grisolía, 1980; Fernández-Solà et al., 2002; Otis and Guidot, 2009). Concomitantly, ROS production in skeletal muscle is increased after chronic EtOH intake, as evidenced by elevated protein carbonylation and lipid peroxidation (Adachi et al., 2003a; Otis et al., 2007; Kumar et al., 2019; Ganjayi et al., 2023). Persistent EtOH-induced ROS production can lead to membrane damage due to lipid modifications (Simon et al., 2023), which in turn stimulates the production of proinflammatory cytokines such as TNF-alpha (Patel and Patel, 2017) and IL-6 (Steiner et al., 2015). These cytokines further increase free radical production and levels of oxidative stress (Otis and Guidot, 2009). Prolonged exposure to ROS and proinflammatory cytokines impairs mitochondrial function (Kumar et al., 2019), favoring metabolic inflexibility. Short-term EtOH consumption has also been shown to alter cholesterol metabolism in skeletal muscle, leading to increased levels of oxysterols (Adachi et al., 2000), which persist even after several months of intake (Adachi et al., 2003b). Oxysterols and cholesterol-derived hydroperoxides found in skeletal muscle following excessive EtOH intake have been suggested to signify perturbations in membrane lipids (Adachi et al., 2000; Fujita et al., 2002), including the mitochondrial membranes. Chronic EtOH exposure in muscle cells, specifically cardiac cells, has been reported to decrease mitochondrial membrane potential, reducing mitochondrial function, mitochondrial content, and fatty acid oxidation (Hwang et al., 2023). Taken together, these data demonstrate how EtOH can directly impact skeletal muscle lipids (Figure 1), such as phospholipids, and influence muscle lipid concentrations by reducing mitochondrial function and content.

Indirectly, chronic EtOH exposure dysregulates the lipid profile of other bodily tissues and organs, which in turn can impact skeletal muscle (Figure 1). EtOH specifically has been shown to cause lipolysis in adipose tissue through EtOH-induced secretion of adipokines and activation of adipose triglyceride, hormone sensitive, and monoglyceride lipases (Kang et al., 2007b; Zhong et al., 2012). This tissue-specific lipolysis causes a surge of excess free fatty acids into the bloodstream, which is seen as dyslipidemia in chronic alcoholics and pre-clinical rodent models of excessive EtOH intake (Kang et al., 2007a; Kema et al., 2015). Free fatty acids and other circulating lipids can then enter skeletal muscle causing lipid accumulation, lipid modifications, and influence cellular metabolism (Rubin et al., 1976; Jensen, 2002; Schwenk et al., 2010; Watt and Hoy, 2012; van Hall, 2015; Lipina and Hundal, 2017). Indeed, high fat feeding plus chronic EtOH intake increased lipid peroxidation in mouse skeletal muscle beyond that of EtOH alone (Ismaeel et al., 2022). Here, we provide an example of an indirect pathway that specifically involves adipose-skeletal muscle crosstalk but also acknowledge, that due to EtOH’s widespread effect, other tissue interactions also exist (e.g., liver-skeletal muscle) (Welch et al., 2020).

## Lipids can cause skeletal muscle atrophy

The relationship between EtOH-induced changes in skeletal muscle lipid and muscle atrophy is complex and incompletely defined. It has been reported that downregulation of cardiolipin synthase (crucial for cardiolipin production) reduces myofiber cross-sectional area, muscle mass, and force in the tibialis anterior muscle of young mice (Yoo et al., 2024). Furthermore, others have demonstrated that reductions in lysophospholipids content (particularly lyso-PC) in young mice made the extensor digitorum longus muscle 20% weaker (Ferrara et al., 2021). Additionally, treating muscle cells with palmitate, which leads to ceramide accumulation, increased expression of pro-atrophic genes expression and reduced protein synthesis rates (Woodworth-Hobbs et al., 2014). Similarly, in *drosophila*, muscle-specific knockdown of phosphatidylserine synthase increased rates of apoptosis and autophagy, reduced muscle mass, and impaired motor function (Kim et al., 2024). Though more correlative, several preclinical studies using denervation, immobilization, and high fat feeding demonstrated that intramyocellular lipids (including triglycerides and diglycerides) increased in skeletal muscle and were associated with changes in muscle mass (Kumar and Sharma, 2009; Vigelsø et al., 2016; Fan and Xiao, 2020; Kimura et al., 2021). In humans, unfavorable changes in muscle PC and phosphatidylethanolamine (PE) content also correlate with reduced insulin sensitivity (Newsom et al., 2016) and age-related declines in muscle size and strength (Hinkley et al., 2020).

Based on the lipid species reported to change due to chronic alcohol consumption, we posit that alcohol-induced alterations in lipid metabolism and transport (e.g., skeletal muscle lipid uptake, storage, and oxidation) result in lipotoxicity, leading to apoptosis. Briefly, changes in lipid metabolism can alter membrane compositions, protein distribution and function, and gene expression. Free fatty acids play vital roles, including energy generation and reserve, components of the cell membrane, and ligands for nuclear receptors (de Vries et al., 1997; Turner et al., 2014; Al Saedi et al., 2022). However, disturbances in fatty acid homeostasis, such as inefficient metabolism or intensified release from storage sites, may result in increased free fatty acid levels, leading to an unfavorable accumulation of intracellular lipids (Figure 1). Cells can adjust to free fatty acid intake to a limited extent, yet prolonged exposure to free fatty acids can become deleterious, impairing mitochondrial function, generating ROS, and producing proinflammatory cytokines. Indeed, overloading cells with palmitate, palmitic acid, or ceramides can cause lipotoxicity and apoptosis in otherwise healthy cells (de Vries et al., 1997; Lin et al., 2012; Shan et al., 2013; Yuan et al., 2013; Zorov et al., 2014; Zhang et al., 2017; Li et al., 2019; Mansuri et al., 2021). In the presence of alcohol, apoptosis has been implicated as a mechanism leading to cellular damage in cardiomyocytes, hepatocytes, endothelial cells, thymocytes, lymphocytes, and neural cells (Ewald and Shao, 1993; Beckemeier and Bora, 1998; Baker et al., 1999; Spyridopoulos et al., 2001; Yin and Ding, 2003; Hwang et al., 2005; Pasala et al., 2015; Jan and Chaudhry, 2019). We are aware of only a few studies that assessed apoptosis in chronic alcohol myopathy (CAM) (Fernández-Solà et al., 2002; FernÁndez-SolÀ et al., 2003; Nakahara et al., 2003). The most well-designed and in-depth was conducted in skeletal muscle biopsies of 30 male high-dose well-nourished chronic alcohol consumers and 12 nonalcoholic controls, with apoptosis being assessed by TUNEL, BAX, and BCL-2 immunohistochemical assays (FernÁndez-SolÀ et al., 2003). Chronic alcoholics had significantly higher apoptotic indices in TUNEL, BAX, and BCL-2 muscle assays, and apoptotic indices were higher in alcoholics with skeletal myopathy compared to those without skeletal myopathy (FernÁndez-SolÀ et al., 2003). It can therefore be speculated that alcohol-induced lipoapoptosis could be occurring in skeletal muscle, causing atrophy and weakness in individuals and animals that consume alcohol chronically. These findings collectively underscore the complex relationship between lipid composition and muscle health and offer valuable insights into underlying mechanisms by which lipids may contribute to alcohol-induced tissue dysfunction.

## Future directions

The current article highlights the significance of lipids as a primary factor influencing CAM. While the existing descriptive data provides valuable insights, it lacks a definitive causal link. To advance our understanding, comprehensive omics studies that focus on the skeletal muscle lipidome are essential. Identifying specific lipids altered by chronic EtOH consumption will pave the way for targeted isolation of lipids that affect muscle size. This approach will enable more focused mechanistic studies to investigate the precise impact of lipids on protein anabolic and catabolic pathways. Such data is crucial for the field, as it extends the literature base beyond what is typically studied in CAM, by aiming to clarify the role of lipids in modulating specific gene and protein alterations.

## Conclusion

We suggest that the development and progression of CAM may be due, in part, to the direct or indirect influence of EtOH on skeletal muscle lipids (Figure 1). Chronic EtOH consumption significantly alters lipid composition and metabolism within skeletal muscle, a fact supported by numerous studies published over several decades. These lipid alterations can significantly impact muscle size and function. Recent research has shown that specific changes in lipids can modulate anabolic and catabolic signaling pathways in conditions such as aging, diabetes, and cancer cachexia (Das et al., 2011; Gilbert, 2021; Al Saedi et al., 2022). In summary, this article posits that lipids play a key role in the pathogenesis of CAM, with further research necessary to substantiate this hypothesis.

## Abbreviations

CAM, chronic alcohol-related myopathy; EtOH, ethanol; PC, phosphatidylcholines; PE, phosphatidylethanolamine; ROS, reactive oxygen species.
